# Relationships between the response of the sweet taste receptor, salivation toward sweeteners, and sweetness intensity

**DOI:** 10.1002/fsn3.2036

**Published:** 2020-12-15

**Authors:** Yuko Kusakabe, Yumiko Shindo, Takayuki Kawai, Mari Maeda‐Yamamoto, Yuji Wada

**Affiliations:** ^1^ Food Research Institute National Agriculture and Food Research Organization (NARO) Tsukuba Japan; ^2^ College of Gastronomy Management Ritsumeikan University Kusatsu Japan

**Keywords:** dose–response relationship, salivation, sweeteners, taste receptor

## Abstract

Sweeteners are widely used in food products, and their sweetness potency is usually evaluated by comparing it with that of sucrose. This, however, has led to confusion as some sweeteners are evaluated based on their maximum value of sweet taste response, while others are evaluated by their threshold value. Here, we aimed to develop a novel nonverbal sweetness evaluation system through the sweet taste signal transduction by comparing the responses of the sweet taste receptor, salivation, taste intensity, and preference among six sweeteners. The hT1r2/hT1r3 sweet taste receptor responses represented the input signal of the sweet taste signal transduction, while salivation, sweet taste intensity, and participants' preferences represented the output signals by the gustatory–salivary reflex, primary gustatory cortex area, and the secondary gustatory cortex, respectively. Our results showed that the sweet taste receptor, sweet intensity, and salivary secretion responses were concentration‐dependent and expressed exponentially. Moreover, the results comparing coefficients showed 15–35 times more sensitivity between the response of hT1r2/hT1r3 and the salivation or the sweet taste intensity in non‐nutrient sweeteners. The preference graph curve was not exponential, suggesting that the sweetener preference was not related to the sweet taste receptor, salivation, or sweet taste intensity. These results may suggest that the sweet taste signal of the non‐nutritive sweeteners might be maintained by taste reception by hT1r2/hT1r3 to taste recognition in the primary gustatory area and that receptor responses and salivation could be used as indicators of sweetness intensity.

## Graphical abstract

Sweet intensity, and salivary secretion responses were concentration‐dependent. Sensitivity differences between taste receptor and intensity ranged from 15 to 35. Sweeteners preference is not correlated with the sweet taste receptor.

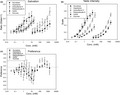

## 1 INTRODUCTION

There are many sweeteners in addition to sugars including, for example, sugar alcohols, amino acids, peptides, proteins, and glycosides (Walters, 2013). In many cases, their sweetness potencies have been evaluated relative to the sweetness of sucrose (O'Donnell & Kearsley, 2012). Generally, the following template is used as an expression of the sweetness quality of the sweeteners. “The target sweetener seems x times sweeter than sucrose.” This representation often leads to the misunderstanding that the ratio represents the magnitude of the intensity of the evaluated sweetener. For example, the so‐called “high‐intensity sweeteners” were considered to elicit a sweet taste even in minute amounts, while their sweetness intensity was not determined. This confusion might have resulted from the comparison of sweeteners against sucrose having been performed (Cameron, 1947) prior to research on the sweet taste receptor and its corresponding signal transduction (Lindemann, 2001).

The sweet taste receptor T1r2/T1r3 has been identified (Nelson et al., 2001), and taste is recognized as a physiological response to chemical stimulation. In this context, the evaluation of sweeteners can be conducted in the same manner as assessments of other chemical substances, such as hormones. The associations between physiological responses and chemical substances are represented as dose–response curves. Taste responses are now expressed using the cells expressing taste receptors; however, few comparisons of dose–response curves for multiple taste substances exist. Additionally, the few reports that have obtained analytical data using psychophysical evaluations represent their findings as dose–response relationships (Antenucci & Hayes, 2015).

In the present study, we aimed to develop a novel nonverbal sweetness evaluation wherein sweetness is defined as taste signal transduction. Specifically, we focused on the input and output processes of taste signal transduction. As an initial study on signal transduction, our aim is to compare the responses of multiple sweet substances to the T1r2/T1r3 receptor response.

The output of taste signal transduction was examined by psychological and physiological methods. We considered salivation as an output of taste signal transduction since it occurs without conscious control, thereby permitting evaluation with unexperienced panelists. Research has revealed two mechanisms of salivation (Hector & Linden, 1999; Mese & Matsuo, 2007): conditioned and unconditioned reflex. The latter accounts for the induction of salivation by taste stimulation. Several comparative analyses have measured the relative extent of salivation in response to the five basic forms of taste stimulation (sweet, bitter, sour, salty, and umami), protein concentration, and pH (Hodson & Linden, 2006; Neyraud et al., 2009). However, there is insufficient information about the relationship between the activity of the taste receptor, taste intensity, and salivation. The relationship between sucrose concentration and salivation has been reported (Hodson & Linden, 2006; Neyraud et al., 2009); however, information about the relationship between multiple sweeteners and salivation has not been reported. Furthermore, there is no study on the relationship between receptor response and the psychophysical sweetness intensity and salivary secretion. Moreover, whether taste‐stimulated salivation includes conditioned reflex salivation, that is, emotional salivation is unclear.

This study therefore compares the responses of T1r2/T1r3, sweet taste intensity as measured by a psychophysical test, and salivation after sweet taste stimulation to evaluate the sweeteners according to their input into and output from the sweet taste signal transduction. Moreover, we compare the participants’ preferences for the sweeteners to explore the relationship among emotion, taste responses by taste receptors, salivation, and taste intensity.

## 2 MATERIALS AND METHODS

### 2.1 Sweeteners

We used six sweeteners in this study: granulated sugar as the sucrose control, acesulfame potassium (MC Food Specialties Inc.), aspartame (Ajinomoto Co.), sucralose (San‐Ei Gen F.F.I.), xylitol (Nippon Garlic Co.), and Rebaudio J‐100 (Morita Kagaku Kogyo Co., LTD.). Rebaudio J‐100 was used as rebaudioside A, because it contains more than 95% rebaudioside A. All solutions were prepared via serial dilutions in Hank's Balanced Salt Solution (HBSS) (Sigma‐Aldrich) for HEK293 cell treatment or in filtered water for psychophysical evaluations by human participants.

### 2.2 Response of the human sweet taste receptors hT1r2 and hT1r3 to sweet‐tasting substances

We used techniques developed previously (Shimizu et al., 2014) to analyze the full‐length human T1r2 and T1r3 [hT1r2/hT1r3] receptor responses to sweet‐tasting substances. Briefly, we used the Flip‐In^™^ 293 cell line (Thermo Fisher Scientific) for the stable expression of hT1r2/hT1r3 and C‐terminal modified G protein, Gα16‐gust44 (Ueda et al., 2003) as sweet taste receptor expressing cells, and hT1r2/hT1r3 cells. Flip‐In^™^ 293 cells expressing only Gα16‐gust44 were used as the control. Ca^2+^ flux assays were performed using a FLEX station 3 (Molecular Devices, LLC) for the cells treated with 100 μl of HBSS containing 5 μM of the calcium indicator dye Fluo8 NW (AAT Bioquest). Stimulation was performed by adding 25 μl of 5X concentrated solutions of sweet‐tasting substances with a pipette. The intensity of the response was recorded as the relative fluorescence units (ΔRFU) value (peak value minus the value for the HBSS) and was plotted against the ligand concentration.

### 2.3 Psychophysical evaluation and saliva collection in human participants

#### 2.3.1 Participants

All experiments were performed according to the standards set by the Declaration of Helsinki and its later amendments. The experimental protocol was approved by the ethics committee at the Food Research Institute, National Agriculture and Food Research Organization, Japan (No.28NFRI‐0003). The experimental procedure was explained to all participants, after which they had the right to cease participation even after initially agreeing to participate in the study. The explanation and the agreement to participate in the experiments were provided in writing. Four men and six women between the ages of 21 and 60 years voluntarily participated in this study (mean age: 37.9 ± 4.0 years). All of them were nonsmokers and in good general health and were not involved in the planning of the taste stimulation procedure.

#### 2.3.2 Procedure

Each session was conducted between 10:00 and 11:00 a.m. or 2:00 and 4:00 p.m. to avoid either a full stomach or hunger. Prior to the study, participants were seated on laboratory chairs in a quiet laboratory. They received instructions about the procedure. After the instructions, a preliminary session was conducted with the participants using water. The procedure was as follows: each participant placed 1 ml of water or a sweet‐tasting solution on his or her tongue using a medicine dropper, kept it in his or her mouth for 10 s, and then swallowed it. After swallowing, saliva was collected for 50 s while the participants recorded their evaluations of the sweetness intensity and their preference for the sweeteners using psychophysical scaling. After the saliva was collected, the participants rinsed their mouths with filtered water and rested for 1 min. The water used for rinsing was swallowed to ensure that the back of the mouth was rinsed entirely. The participants confirmed that the sweet taste had disappeared and then sampled the next solution. Each session included the sampling of 7–9 solutions and lasted for less than 30 min. There were six sessions in total, and no more than one session was conducted per half‐day. Increasing concentrations of sweet‐tasting substances in filtered water were presented during each session. Sucrose was tested first so that it could be compared with the other sweeteners. The order of the sessions was as follows: sucrose, aspartame, rebaudioside A, sucralose, acesulfame K, and xylitol. Participants were not informed of the names of the sweeteners provided, other than sucrose.

#### 2.3.3 Taste stimuli

All taste solutions were prepared using filtered water. The highest and lowest concentrations of the solutions and the number of twofold serial dilution steps are shown in Table 1. Water without taste substances was used as the control. The concentrations of the solutions and the numbers of dilution steps required were investigated in preliminary experiments conducted by the authors (data not shown). The solutions were presented in 1‐mL volumes in 2‐mL tubes at 25°C. They were stored in a freezer; as an exception, the aspartame solutions were prepared within 4 hr of the sessions.

**TABLE 1:** Sweetener solutions by twofold serial dilutions

	Taste receptor response			Taste intensity, salivation, preference		
	Highest (mM)	Lowest (mM)	Number of solutions	Highest (mM)	Lowest (mM)	Number of solutions
Sucralose	5	0.000610	14	10	0.0781	8
Aspartame	5	0.000610	14	20	0.156	8
Acesulfame K	8	0.000977	14	16	0.25	7
Rebaudioside A	2	0.000244	14	1	0.0313	6
Xylitol	400	0.0488	14	1,600	12.5	8
Sucrose	400	0.0488	14	1,600	12.5	8

#### 2.3.4 Saliva collection

We collected the saliva in 2‐oz plastic cups. The amount of saliva was determined by weighing it on a scale. We weighed the saliva secreted following water stimulation before the testing of the sweeteners began, and this weight was used as a control. The change in salivation was evaluated by calculating the ratio of the weight of saliva secreted after the sweet stimulation to that after the water stimulation.

#### 2.3.5 Psychophysical scaling

A generalized labeled magnitude scale (gLMS; Green et al., 1993) was used to measure sweetness intensity. As previously reported (Green et al., 1993), the gLMS ranged from zero (no sensation), 1.4 (barely detectable), 6 (weak), 17 (moderate), 35 (strong), 51 (very strong), to 100 (strongest imaginable sensation of any kind). We analyzed the participants' preferences for the sweeteners using a structured line scale. The line was 196 mm in length and ranged from −1 (dislike), through 0 (midpoint), to 1 (like; Kemp et al., 2009).

### 2.4 Statistical processing

#### 2.4.1 Concentration–Response graph

All data were plotted on graphs. The log of the sweetener concentrations was plotted on the *x*‐axis. Different parameters were plotted on the *y*‐axis of the graph as follows: the intensity of the response of the hT1r2/hT1r3 cells (ΔRFU) and the relative amount of saliva secreted was plotted arithmetically; the sweetness intensity measured using the gLMS and the sweet preference on a structured line scale were measured using rulers and were plotted arithmetically. The intensity of the response of the hT1r2/hT1r3 cells was fitted to the Hill equation (*y* = *ax^b^*/(*c^b^* + *x^b^*)), and the half‐maximal effective concentrations (EC_50_) were calculated using SigmaPlot software (Systat Inc.). The graphs of the response of the hT1r2/hT1r3 cells, the relative amount of saliva secreted, and the sweetness intensity were fitted to one of the exponential functions (exponential rise to maximum, simple exponent, or three Parameter) using SigmaPlot software. The formula is *y* = *y*_0_ + *a*(1 – *e* ^−^ *^bx^*).

#### 2.4.2 The sample correlation values

The sample correlation values (*r*) were calculated as follows:

$$r=\frac{\left(n\sum \mathrm{xy}‐\left(\sum x\right)\left(\sum y\right)\right)}{{\left(n\left(\sum x^{2}\right)‐{\left(\sum x\right)}^{2}\right)}^{1/2}{\left(n\left(\sum y^{2}\right)‐{\left(\sum y\right)}^{2}\right)}^{1/2}}$$

Significant differences were determined using *t* tests. Probability values for the correlation were calculated from a t distribution with (*n*−2) degrees of freedom as follows:

$$t=\frac{r\left(n‐2\right)1/2}{\left(1‐r2\right)1/2}$$

We used Microsoft Excel for calculations.

## 3 RESULTS

### 3.1 Response of the sweet taste receptor hT1r2/hT1r3 in HEK293 cells to sweeteners

**FIGURE 1:**
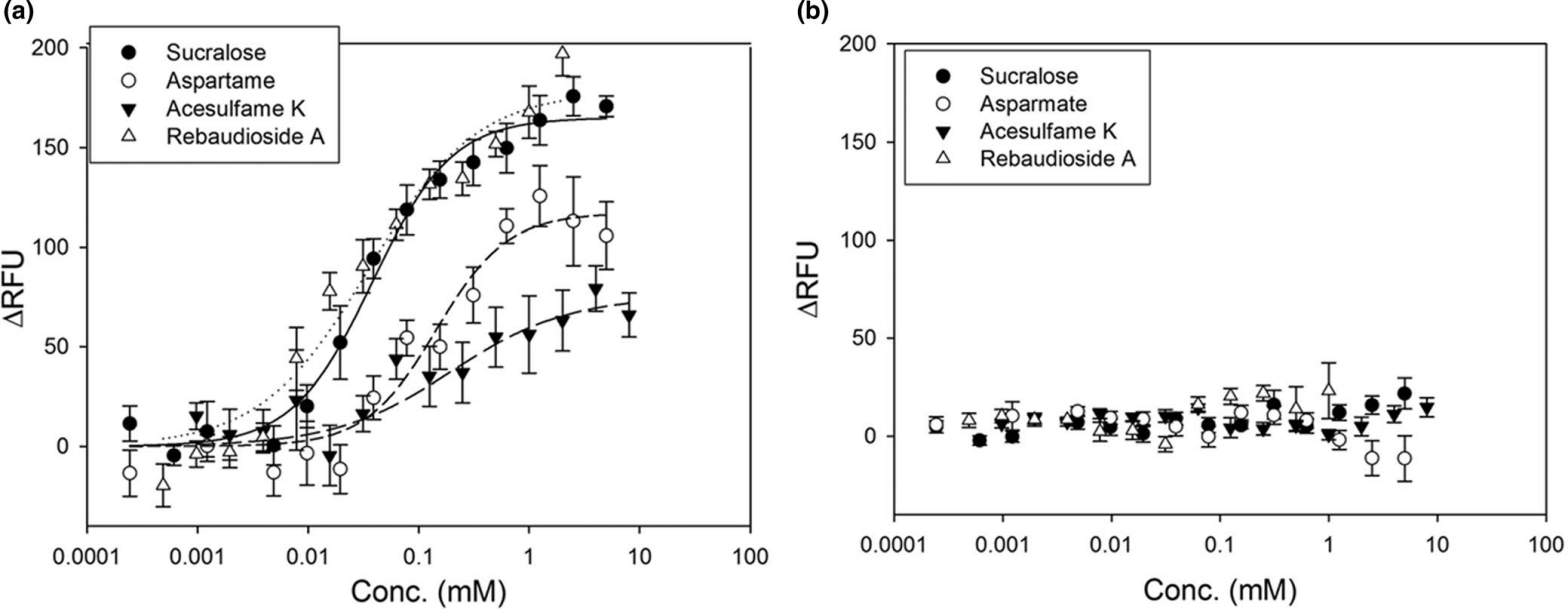
Concentration–response relationship of human sweet taste receptor with sweeteners. The intensity of the responses of hT1r2/hT1r3 cells (a) and control cells (b) represented as elevations of intracellular Ca^2+^ elevation. The relative fluorescence units (ΔRFU) were plotted against the sweetener concentrations. Error bars: *SD* (*n* = 4–22). The graphs were fitted to the Hill equation (*y* = *ax^b^*/(*c^b^* + *x^b^*)) (a)

We first analyzed the response of the human sweet taste receptor, G protein‐expressing cells (hT1r2/hT1r3 cells), and G protein solely expressing cells (control cells) to the selected sweeteners using a Ca^2+^ imaging technique. The responses to the sweeteners are displayed in Figure 1. The graph shows a concentration–response relationship, and it was fitted by Hill's equation: *y* = *ax^b^*/(*c^b^* + *x^b^*) (*R* ^2^ = .947–.987), where “*a*” is the estimated maximum value. The order was rebaudioside A > sucralose > aspartame > acesulfame K (Table 2). Variable “*b*” indicates the slope and “*c*” is the EC_50_ value (mM). The EC_50_ values of aspartame and sucralose are almost the same as those reported previously (Li et al., 2002; Servant et al., 2010; Zhang et al., 2010). We were unable to adequately measure the response to xylitol and sucrose. The responses to xylitol and sucrose decreased above 10 mM, and large responses to 400 mM of sucrose and xylitol were observed, in contrast to previous reports (Li et al., 2002; Servant et al., 2010; Zhang et al., 2010; Figure S1). Therefore, we removed the responses of xylitol and sucrose from this comparison.

**TABLE 2:** Comparison of graph shapes for hT1r2/hT1r3 response via coefficients by Hill equation

	Sucralose	Aspartame	Acesulfame K	Rebaudioside A
A	165	117	74.0	178
B	1.19	1.36	0.882	0.890
C	0.0382	0.145	0.191	0.0354

Note

The graph formula: Hill equation: *y* = *ax^b^*/(*c^b^* + *x^b^*).

*a*: estimated maximum value, *b*: slope, *c*: EC_50_ (mM).

### 3.2 The relationship between the concentration of the sweeteners and salivation, taste intensity, and preference

**FIGURE 2:**
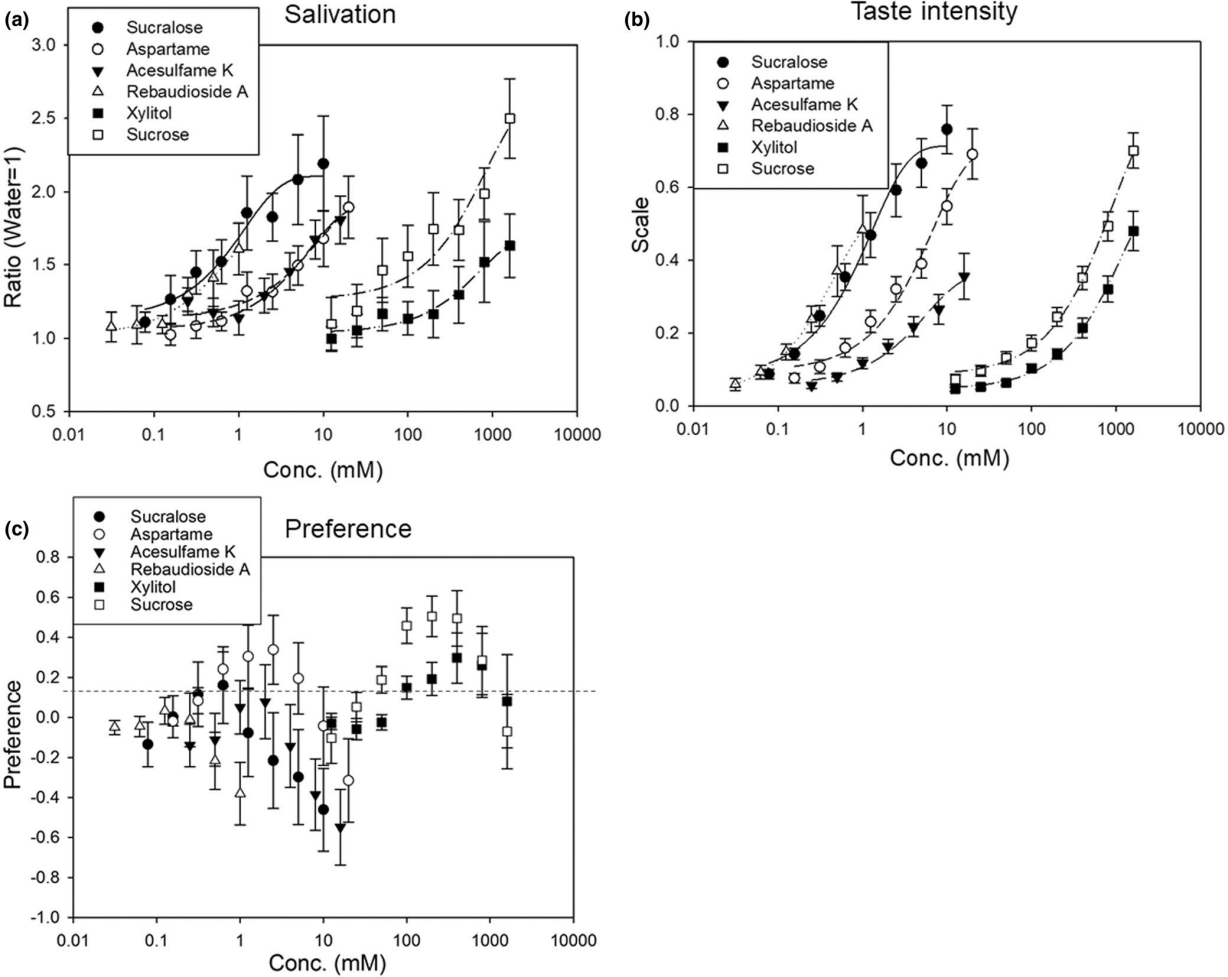
Concentration–response of sweeteners as determined by psychophysical evaluation and salivation. (a) Salivation in response to sweeteners. The ratio is relative to the amount of salivation induced by stimulation with water (*n* = 10). (b) The intensity of sweeteners as determined by the psychophysical evaluation. Representation of the intensity is based on the gLMS scale (score: 0–100; *n* = 10). The graphs (a, b) were fitted to the exponential function *y* = *y*_0_+*a* (1 − *b^x^*). (c) Participants' preferences for sweeteners. The degree of preference is represented by a nonstructured line (score: −1 to +1; *n* = 10)

In a preliminary experiment (data not shown [*n* = 3]) performed before the analysis, the concentrations of the sweetener that could be clearly recognized by the participants were elucidated. As high concentrations of the sweeteners resulted in a bitter taste, they were avoided. In particular, as rebaudioside A tasted bitter at a concentration of 2 mM, no data above this concentration were obtained. We measured the amount of salivation using the spit method. Every sweetener resulted in increased salivation as the sweetness concentration increased (Figure 2a). The relationship between the concentration of sweetener and the amount of saliva secreted differed for each sweetener. The graph of the relationship between the concentration of sweetener and the amount of saliva was fitted to one of the exponential functions: *y* = *y*_0_ + *a* (1 – *e* ^−^ *^bx^*) (*R* ^2^ = .860–.961): “*a*” indicates the estimated maximum value (the descending of which was sucrose > sucralose > aspartame > acesulfame K > rebaudioside A > xylitol [Table 3A]), “*b*” is sensitivity of the substances (the descending order of which was rebaudioside A > sucralose > aspartame > acesulfame K > xylitol > sucrose [Table 3A]).

**TABLE 3:** Comparison of graph shapes for hT1r2/hT1r3 response, salivation, and sweet taste intensity via coefficients

A) Salivation						
	Sucralose	Aspartame	Acesulfame K	Rebaudioside A	Sucrose	Xylitol
a	0.974	0.859	0.796	0.758	1.374	0.683
b	0.845	0.147	0.124	1.497	0.0012	0.0014

B) Sweet taste intensity						
	Sucralose	Aspartame	Acesulfame K	Rebaudioside A	Sucrose	Xylitol
a	0.634	0.616	0.306	0.5193	0.737	0.589
b	0.772	0.148	0.124	2.139	0.0011	0.0008

C) hT1r2/hT1r3 response				
	Sucralose	Aspartame	Acesulfame K	Rebaudioside A
a	158.7	121.0	54.74	164.1
b	18.55	5.038	3.517	31.79

D) Estimated sensitivity difference: The ratio of “*b*” values						
	Sucralose	Aspartame	Acesulfame K	Rebaudioside A	Sucrose	Xylitol
Receptor/Salivation	24.03	34.13	28.36	14.86	N.D.	N.D.
Receptor/Intensity	21.95	34.18	28.36	21.24	N.D.	N.D.
Salivation/Intensity	1.095	0.9986	1.000	0.6998	1.090	1.750

Note

The graph formula: *y* = *y*_0_ + *a*(1 − *e* ^−^ *^bx^*).

*a*: estimated maximum value, *b*: value of sensitivity.

We conducted a psychophysical measurement while measuring salivation and evaluated the sweetness intensity using the gLMS method. As observed for salivation, the sweetness intensity of every sweetener increased with an increase in sweetness concentration (Figure 2b). The graph of the relationship between the concentration of the sweetener and the sweetness intensity was also fitted to *y* = *y*_0_ + *a* (1 – *e* ^−^ *^bx^*) (*R* ^2^ = .980–.999; Table 3B). The order of the estimated maximum value “*a*” was sucrose > sucralose > aspartame > xylitol > rebaudioside A > acesulfame K. The order of the sensitivity of the substances “b” was rebaudioside A > sucralose > acesulfame K > aspartame > sucrose > xylitol.

We analyzed the participants' preferences for the sweet solutions while the sweetness intensity was being evaluated (Figure 2c). Each participants' preferences varied considerably, although they largely tended to decrease with increasing concentrations. This graph was not fitted to any formula.

### 3.3 Correlations between responses of the sweet taste receptor, salivation, and sweet taste intensity

We attempted to compare our dose–response graphs, which had different vertical axes and shapes. We found that the response graph for T1r2/T1r3 was also fitted to the equation; *y* = *y*_0_ + *a* (1 – *e* ^−^ *^bx^*) (*R* ^2^ = .849–.975; Table 3C). The order of the values “*a*” were rebaudioside A > sucralose > aspartame > acesulfame K, supporting the maximum response value results by Hill's equation (Table 2). The value of “*x*” when *e* ^−^ *^bx^* was 0.5, which also corresponded with the value of EC_50_ seen in Table 2. We therefore considered the ratio of the values of “*b*” to represent the differences in sensitivity between responses of the sweet taste receptor and the salivation and the sweet taste intensity (Table 3D). The ratio value varied depending on the sweetener, which had a range of 15–35 between the sweet taste receptor and salivation, and about 20–35 between the sweet taste receptor and the sweet taste intensity. The ratio between the salivation and the sweet taste intensity was relatively the same (0.7–1.75; Table 3D). We next calculated the correlation coefficients using the mean values of non‐nutrient sweeteners since the response data of hT1r2/hT1r3 did not correspond to the personal psychophysical evaluation or physiological response data.

We compared the results of the sweet taste receptors using 1/32 of the original concentrations for sweet taste intensity, salivation, and participants' preferences. The concentration of 1/32 was determined in consideration of the difference in sensitivity and the method of preparing the solution: the differences of the sensitivity ranged from 15 to 35 between the responses of hT1r2/hT1r3 (Table 3D). The solutions were prepared with twofold serial dilutions for the response analysis (Table 1). All correlation coefficients values between the sweet taste receptor response, the salivation, and the sweet taste intensity were high (*r* > .9, Table 4). The relationship between salivation, sweet taste intensity, and preference for the sweeteners including sucrose and xylitol was analyzed individually. The correlation coefficients were lower when the individual values were used rather than the mean values (Table 5); however, a significant correlation was observed between taste intensity and salivation. For each sweetener, except for rebaudioside A, the correlation coefficients for sweetness intensity and salivation were significant. Inverse correlations were observed between the mean and individual data for the participants’ preferences and the response of hT1r2/hT1r3 (Table 5). Using the individual values, a significant inverse correlation was observed between the participants' preference and salivation for all the sweeteners but sucrose. A significant inverse correlation was observed between participants’ preference and sweetness intensity except for sucralose, aspartame, and rebaudioside A (Table 5).

**TABLE 4:** Correlations among the taste receptor, taste intensity, and salivation responses (mean values)

	Taste receptor response (1/32th conc. of non‐nutritive sweeteners)	Taste intensity	Salivation
Taste intensity	.952**		
Salivation	.910**	.904**	
Preference	−.445*	−.314 (*p* = .35)	−.302*

* *p* < .05.

** *p* < .001.

**TABLE 5:** Correlations among salivation, sweet taste intensity, and preference for sweeteners (individual values)

	Taste intensity versus Salivation	Taste intensity versus Preference	Salivation versus Preference
Sucralose (*n* = 72)	.521**	−.432**	−.247**
Aspartame (*n* = 72)	.685**	−.381**	−.373**
Acesulfame K (*n* = 63)	.447**	.075 (*p* = .535)	−.480**
Rebaudioside A (*n* = 54)	.204 (*p* = .118)	−.533**	−.319*
Xylitol (*n* = 72)	.556**	.074 (*p* = .514)	−.230*
Sucrose (*n* = 72)	.597**	−.0095 (*p* = .934)	.028 (*p* = .805)
Total (*n* = 450)	.539**	−.211**	−.247**

* *p* < .01.

** *p* < .001.

## 4 DISCUSSION

We compared the response of human sweet taste receptors in cultured cells, taste intensity, salivation, and subject preferences in response to six sweeteners. We found that the response of the sweet taste receptor, sweet taste intensity, and salivation volumes correlated with each other for non‐nutritive sweeteners. This study is, to the best of our knowledge, the first to have performed a multilateral analysis of a range of sweeteners using molecular, cellular, biological, sensory, and physiological evaluation methods.

This study showed that all the responses to the sweet taste receptor, the sweet intensity, and the salivation to the sweeteners can be characterized as dose–response results. As Antenucci and Hayes (2015) indicated previously, earlier representations of sweeteners’ properties have confused the strengths of the response with binding strength or potency. Several reports have previously demonstrated the response of cells expressing hT1r2/hT1r3 to each of the sweet‐tasting substances investigated here (Masuda et al., 2012; Servant et al., 2010; Xu et al., 2004). However, these reports documented the response of these cells to each sweetener separately and did not establish their correlation with salivation or psychophysical evaluation. Hence, presenting the dose responses of multiple sweeteners in a single graph and comparing the different lines of evaluation in this study were unprecedented. However, a simple comparison of these responses was difficult because the concentrations of the sweeteners were always represented on their horizontal axes while the parameters represented on the longitudinal axes were not uniform. All graphs were represented by exponential functions, so comparing the coefficients and clarifying the differences in sensitivity between the analysis was possible.

Various factors may account for the differences. First, the sweet solutions were naturally diluted at T1r2/T1r3 on the taste cells in the oral cavity because of the presence of saliva and mucus around the taste cells. We used 1 ml of solution for salivation and psychophysical evaluation to reduce the burden on the participants and examine multiple samples in a short period of time. This volume is lower than that used in the whole mouth method (Yamauchi et al., 2002), which results in higher dilution in the oral cavity. However, the 1‐mL solution was useful for continuous stimulation at short intervals because the sweet taste dissipates in less time. Second, the signal transduction pathway of the T1r2/T1r3 receptors on HEK293 cells differs from those found on taste cells. For example, the G protein bound to T1r2/T1r3 in HEK293 is Galpha16‐gust, which is artificial; the analogous protein in taste cells has not been identified.

Non‐nutritive sweeteners were less palatable, allowing us to distinguish whether the salivation was due to the intensity of sweetness or due to palatability; the low preference of non‐nutritive sweeteners was most likely due to a bitter and metallic taste (Schiffman et al., 1985). In our study, the non‐nutritive sweeteners were used at a concentration that did not exhibit bitterness, but it might be possible that they bound to the bitterness receptor and induced unpleasantness despite there being no awareness of bitterness. Our observation that adjusting the sensitivity could yield high correlations suggested that the sweet taste signals were transduced while maintaining their relative relationships with the binding responses of T1r2/T1r3 to recognition in the primary gustatory cortex through the salivary nucleus. We did not observe that the palatability of the sweeteners was related to the salivation. This finding supports the theory that salivation caused by taste stimulation is attributable to an unconscious reflex because the unconscious salivation induced by sweeteners might be independent of emotion. Saliva is secreted conditionally or unconditionally (Hector & Linden, 1999; Mese & Matsuo, 2007), and conditional secretion following taste stimulation is a gustatory–salivary reflex that is mediated by the nucleus of the solitary tract and salivatory nucleus. Conditional secretion occurs in response to emotional triggers (Ekström et al., 2011). In our study, the responses of hT1r2/hT1r3, sweetness intensity, and the salivation due to non‐nutritive sweeteners were found to be associated. In addition, the sweetness intensity and salivation were not influenced by the preference for the sweeteners. Hence, considering the stimulation of taste alone, saliva may be secreted in an unconditional manner. Taste signals are thought to be transmitted via taste nerves, the nucleus of the solitary tract, the primary gustatory cortex, and the secondary gustatory cortex (Rolls, 2005). The primary gustatory cortex relays the taste quality and intensity, while the secondary gustatory cortex relays the total preference for the food. Taking this into account, our results raise the possibility that the response profile of the non‐nutritive sweeteners might be conserved from the taste receptor to the primary gustatory cortex.

There are several limitations to our study. First, the number of participants was limited. The difference in the descending order of the estimated maximum value and sensitivity of the substances for salivation and taste intensity might not be observed if there were more participants. On the other hand, it is unlikely that the relationship between cell responses, salivation, and intensity had a high correlation by chance since the names of the sweeteners were not given to the participants, except for sucrose which is the standard sweetener, thereby preventing potential bias with information that might affect taste intensity or salivation. Moreover, salivation is not controlled intentionally.

Second, we needed to present increasing concentrations of sweet solutions during each session in this study. With our method, the sweetness disappeared when the lower concentration solutions were tasted after the higher concentration solutions. This was probably due to desensitization. To eliminate desensitization to the continuous sweetness stimuli, our protocol of making sure the sweetness disappeared by resting for a minute after drinking water to cleanse the palate may have been insufficient. A method to eliminate desensitization in a short time with less burden on the subjects needs to be investigated in the future.

Third, the responses of sucrose and xylitol could not be analyzed by our heterologous expression system. We speculated that cells have a high sensitivity toward osmotic pressure induced by high concentrations of sugar or xylitol; hence, we were unable to measure the responses of hT1r2/hT1r3. However, several studies have demonstrated the dose‐relationship of sucrose using hT1r2/hT1r3 in HEK293 or HEK293T cells (Galindo‐Cuspinera et al., 2006; Jiang et al., 2004). This problem may have been particular to our HEK293 cells. The second limitation is the individual differences in sweet taste sensitivity to rebaudioside A. The differences might be due to genetic factors or physiological conditions. Further studies are needed to clarify this.

Finally, there are many methods by which to measure the salivary flow rate, such as suction, drainage, or cotton wool rolls (Navazesh, 1993). In our study, we used the spit method to minimize the stress imposed on the participants. However, while the spit method is minimally burdensome, we were unable to identify the specific salivary gland that secreted the saliva. Other studies have shown that saliva caused by taste stimulation is secreted from the parotid gland (Hodson & Linden, 2006; Neyraud et al., 2009); however, we could not confirm the origin of the saliva. While this limitation results from the negotiation between accuracy and simplicity, future research should a devise a method that allows for high degrees of both.

This study succeeded in elucidating the relationship between the input of the sweet taste signal to the sweet taste receptor hT1r2/hT1r3 and to both the physiological output of salivation and to the recognition of sweetness intensity. We found that the sweet taste responses in the hT1r2/hT1r3 expressed on HEK293 cells correlated with the levels of unconscious salivation and with taste intensity of the human participants. Moreover, our correlation analysis clarified sensitivity differences, introducing the possibility that the sweetness intensity of non‐nutritive sweeteners can be evaluated by measuring salivation and the responses of hT1r2/T1r3 receptors. Therefore, untrained persons could evaluate single tastes without bias and complete global evaluations without language. Future research should devise a method by which to elucidate the relationship between conscious and unconscious food sensing systems, as this would help to gain insight into individual palatability.

## CONFLICT OF INTEREST

The authors declare that they do not have any conflict of interest.

## ETHICAL APPROVAL

This study was approved by the ethics committee at the Food Research Institute, National Agriculture and Food Research Organization, Japan (No. 28NFRI‐0003).

## INFORMED CONSENT

Written informed consent was obtained from all study participants.

## Supporting information

Fig S1
