# Supplementary figures and images for: Relationships between the response of the sweet taste receptor, salivation toward sweeteners, and sweetness intensity
Source: Food Sci Nutr. 2020 Dec 15;9(2):719–27. doi: 10.1002/fsn3.2036 (PMC7866590; doi:10.1002/fsn3.2036)

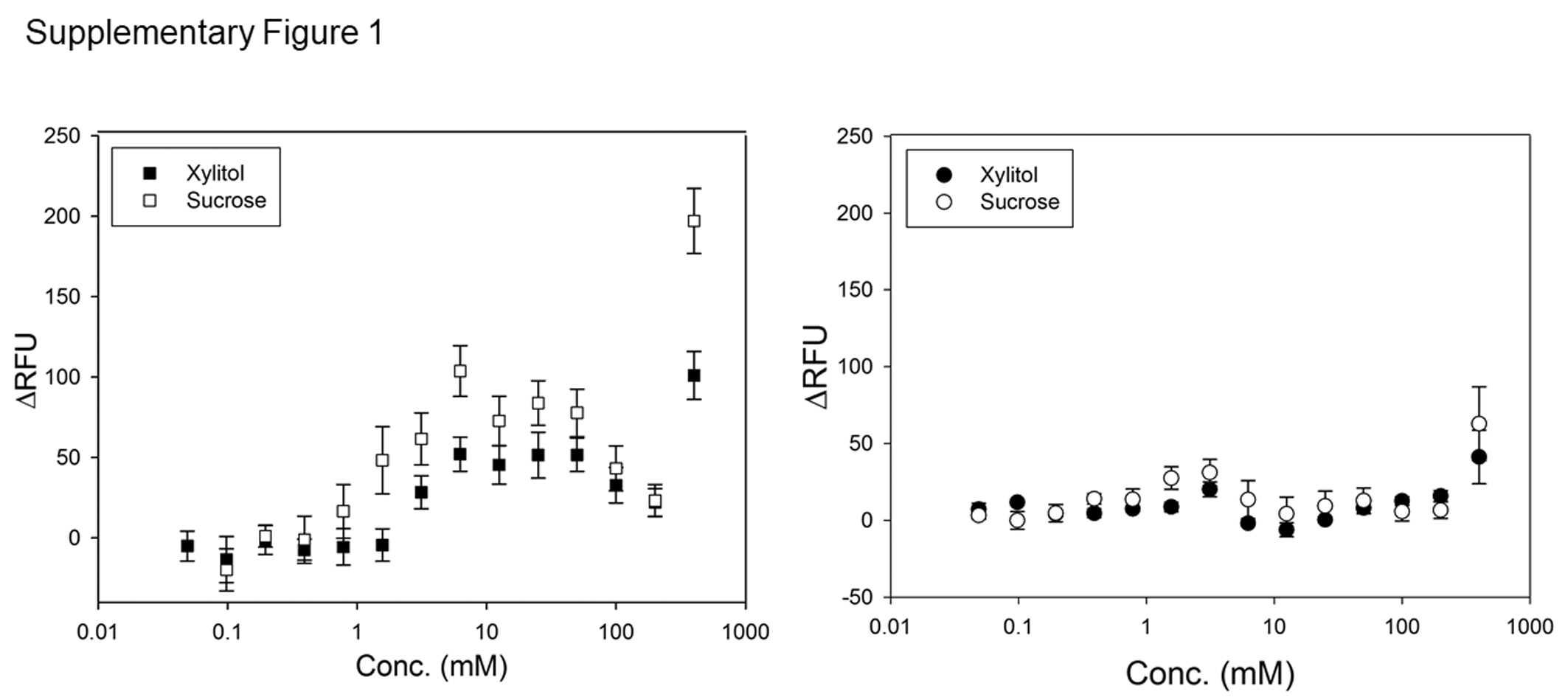

Supplement: Supplementary file 1 — Fig S1 [file FSN3-9-719-s001.tif]
